# Binding of a Pyrene-Based Fluorescent Amyloid Ligand to Transthyretin: A Combined Crystallographic and Molecular Dynamics Study

**DOI:** 10.1021/acs.jpcb.3c02147

**Published:** 2023-07-21

**Authors:** Nghia Nguyen Thi Minh, Afshan Begum, Jun Zhang, Petter Leira, Yogesh Todarwal, Mathieu Linares, Patrick Norman, Dean Derbyshire, Eleonore von Castelmur, Mikael Lindgren, Per Hammarström, Carolin König

**Affiliations:** † Institute of Physical Chemistry and Electrochemistry, Leibniz University Hannover, Callinstr. 3A, 30167 Hannover, Germany; ‡ Division of Chemistry Department of Physics, Chemistry and Biology, Linköping University, 581 83 Linköping, Sweden; § Department of Physics, Norwegian University of Science and Technology, 7491 Trondheim, Norway; ∥ Department of Theoretical Chemistry and Biology, School of Engineering Sciences in Chemistry, Biotechnology and Health, KTH Royal Institute of Technology, SE-106 91 Stockholm, Sweden; ⊥ Laboratory of Organic Electronics, ITN, Linköping University, PSE-581 83 Linköping, Sweden; # Scientific Visualization Group, ITN, Linköping University, SE-581 83 Linköping, Sweden

## Abstract

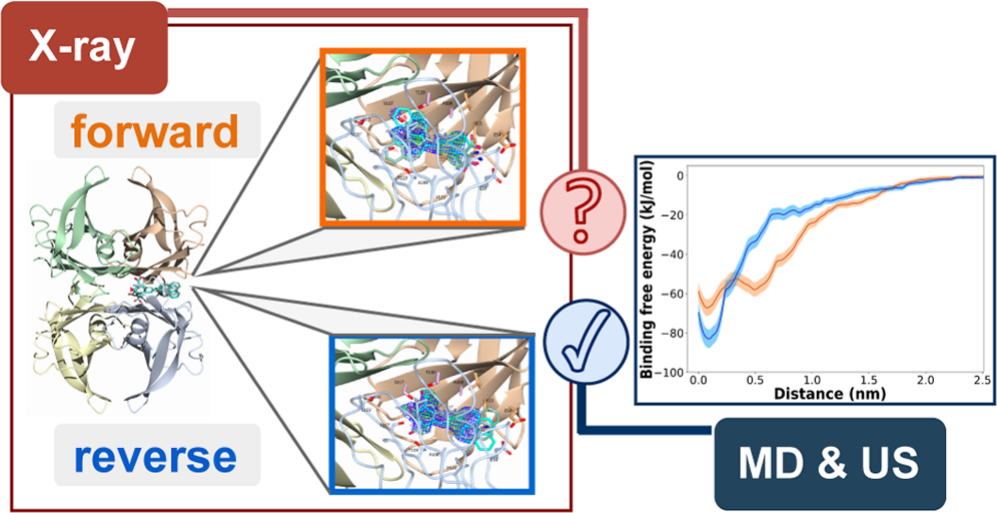

Misfolding and aggregation of transthyretin (TTR) cause several amyloid diseases. Besides being an amyloidogenic protein, TTR has an affinity for bicyclic small-molecule ligands in its thyroxine (T4) binding site. One class of TTR ligands are trans-stilbenes. The trans-stilbene scaffold is also widely applied for amyloid fibril-specific ligands used as fluorescence probes and as positron emission tomography tracers for amyloid detection and diagnosis of amyloidosis. We have shown that native tetrameric TTR binds to amyloid ligands based on the trans-stilbene scaffold providing a platform for the determination of high-resolution structures of these important molecules bound to protein. In this study, we provide spectroscopic evidence of binding and X-ray crystallographic structure data on tetrameric TTR complex with the fluorescent salicylic acid-based pyrene amyloid ligand (Py1SA), an analogue of the Congo red analogue X-34. The ambiguous electron density from the X-ray diffraction, however, did not permit Py1SA placement with enough confidence likely due to partial ligand occupancy. Instead, the preferred orientation of the Py1SA ligand in the binding pocket was determined by molecular dynamics and umbrella sampling approaches. We find a distinct preference for the binding modes with the salicylic acid group pointing into the pocket and the pyrene moiety outward to the opening of the T4 binding site. Our work provides insight into TTR binding mode preference for trans-stilbene salicylic acid derivatives as well as a framework for determining structures of TTR–ligand complexes.

## Introduction

Transthyretin (TTR) is a 55 kDa homotetrameric secreted protein with 127 amino acids in each subunit. TTR is synthesized mainly by the liver and the choroid plexus, and to some extent in the pancreas and the eye.^1^ It is an important protein for metabolic homeostasis and has also been suggested to function as a molecular chaperone to prevent aggregation of proteins associated with neurodegenerative diseases.^2^ TTR circulates in human plasma and cerebrospinal fluid and functions as a transport protein of the metabolic hormone thyroxine (T4) and retinol (Vitamin A) through complex formation with retinol-binding protein (RBP).^1^ T4 binds directly to TTR. The T4 binding site is characterized by three subsites, each composed of pairs of symmetric and hydrophobic halogen binding pockets (HBPs). HBP 1 and 1′ are in the outer cavity; HBP 3 and 3′ are the inner binding subsite; and HBP 2 and 2′ are the intervening interfaces between them.^3,4^ Hydrogen bonding to the bound ligand is enabled through Ser-117 and Thr-119 buried at the bottom of the binding site. The entrance to the binding site is flanked by opposing Lys15 residues. In elderly individuals, wild-type TTR misfolds and aggregates into amyloid fibrils mainly manifesting as cardiac amyloidosis.^5^ There are also numerous familial forms of TTR amyloid disease caused by over 140 point mutations in the TTR gene often causing familial amyloid polyneuropathy (FAP).^5^ Point mutations are inherited dominantly and cause production of a destabilized tetrameric TTR protein, elevating the risk for amyloidosis. There are several treatment options for TTR amyloid diseases including liver transplantation, which surgically removes the liver-produced familial TTR protein.^6^ Transcription downregulation can be achieved using small interfering RNA (siRNA) and antisense oligonucleotides (ASOs).^7^ Small-molecule stabilizers can kinetically stabilize the TTR tetramer, thereby reducing protein misfolding.^8^ A first clinical trial of CRISPR/Cas9 has recently been performed with successful results in the reduction of TTR production in humans.^9^ Currently, work is ongoing to better diagnose patients early, to follow up and monitor the various available treatments, and to find improved small-molecule ligands as kinetic stabilizers.^10−14^

We are using TTR as a research platform to understand small-molecule recognition and binding specificity in our work to facilitate these efforts.^15,16^ While the structure of TTR was originally solved in 1978^17^ and hundreds of structures of TTR variants and TTR complexes are present in the protein database (PDB), the binding modes of TTR ligands are still a matter of intense research: Recently, a quinoline-derived D–A–D-type fluorescent probe was utilized by Sun et al. to study its binding to wild-type TTR,^18^ while previous work by some of us^19^ has identified a pyrene-based trans-stilbene ligand with a salicylic acid moiety (Py1SA) as an amyloid fibril probe for several different amyloid proteins. Py1SA is an amyloid fluorophore containing a combination of a salicylic acid trans-stilbene conjugated with a pyrene.^19^ Its design was based on the pan-amyloid Congo red analogue X-34 and the pyrene moiety from its extensive fluorescence lifetime. The spectroscopic features of Py1SA with a variety of solvents were reported previously in the context of amyloid binding.^19^ Excited-state intramolecular proton transfer along with intramolecular charge transfer was observed for the anionic form in polar solvents.^19^

In the present work, we reveal that Py1SA can in addition to A*β*1-42 fibrils^19^ also bind to the native state of TTR through its T4 binding site. We have previously observed that trans-stilbene-based small molecules bind to the TTR T4 binding site,^20,21^ but a compound comprising a pyrene was rather surprising due to its bulkiness. The crystallographic data of the complex, however, do not allow unambiguously demonstrating the binding mode, mainly due to partial occupancy. Consequently, the most likely orientation of the ligand in the binding pocket could not be resolved experimentally. We, therefore, complement the experimental study with molecular dynamics and umbrella sampling calculations that give clear evidence for a dominant orientation of the ligand and thereby describe the TTR–Py1SA complex at atomic resolution.

## Methods

The photophysical properties of typically 1–4 μM TTR and Py1SA solutions in phosphate-buffered saline (PBS) were measured using steady-state and time-resolved fluorescence spectroscopy at room temperature (20 °C) and similar procedures as in earlier work,^22,23^ see also Section S-1.1 in the Supporting Information (SI). The expression and purification of human TTR as well as the crystallization were carried out as described previously^24^ and are briefly summarized in Sections S-1.2 and S-1.3 in the SI. The X-ray diffraction (XRD) data were collected under cryogenic conditions at the MAX VI facility, Sweden, and processed to a resolution of 1.4 Å.^25,26^ Phasing was done by molecular replacement.^27^ The search model was derived from the published coordinates 1F41, omitting terminal residues and a known flexible region. Hence, the protein structure was determined for residues 11–98 and 104–122 within each monomer. It was refined against the diffraction data^28^ including manual map inspection.^29^ For more details on the X-ray analysis, we refer to Section S-1.4 in the SI.

For the molecular dynamics (MD) simulations, we applied Gromacs 2019.3^30^ with the Amber ff14SB force field^31^ for the TTR protein, TIP3P^32^ for water, and a General Amber Force Field (GAFF)^33,34^ re-parametrized for Py1SA against B3LYP data (see Section S-1.5 in the SI). The re-parameterization procedure has been described in earlier work.^35^ As initial structures, we applied refined (but ambiguous) structures from the X-ray analysis after the required preprocessing.^36,37^ In these structures, the ligand is located in the binding pocket. After the optimization step, all protein atoms, except the ones that are within 4 Å of the binding pocket, as well as all bonds have been constrained in the further MD simulations. For each of the four equilibrated structures, we conducted a 1 μs MD simulation in the NVT ensemble at 300 K, i.e., under ambient conditions (see also Section S-1.6 in the SI). The umbrella sampling (US)^38−40^ simulations were carried out with Gromacs version 2021.3. As coordinate, we choose the center-of-mass (COM) distance between the pyrene or benzene group of the Py1SA ligand and the binding pocket starting from the TTR–Py1SA complex for the reverse and forward binding modes, respectively. Here, the binding pocket is defined by all atoms within 4 Å from the ligand in the equilibrated structure. In the US simulations, we constrained all backbone movement. Further information on the US details can be found in Section S-1.7 in the SI.

## Results and Discussion

### Spectroscopic Evidence for Py1SA Ligand Binding to TTR

**Figure 1:**
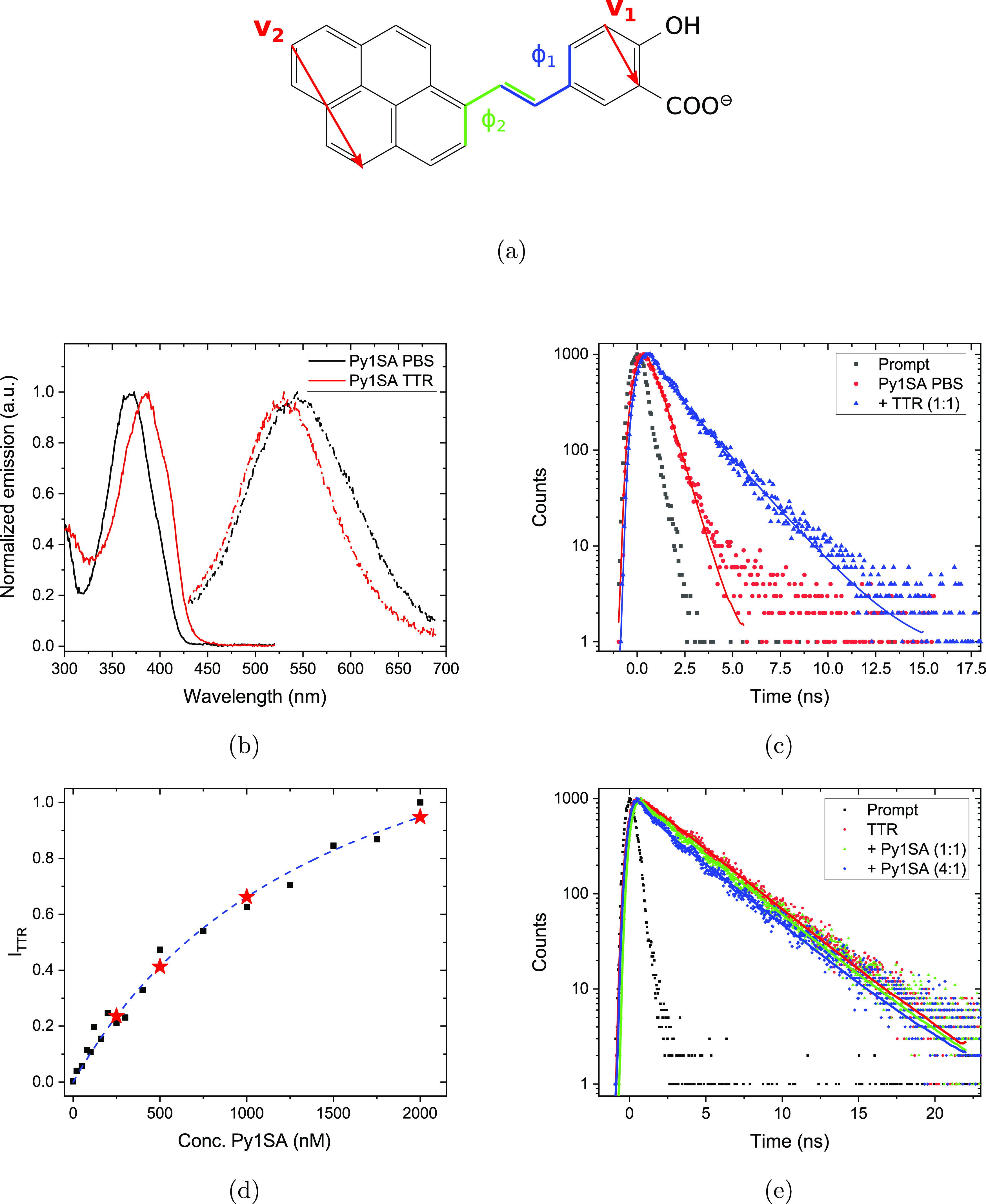
Photophysical properties of the Py1SA fluorescent ligand in PBS with and without the presence of TTR. (a) Schematic representation of Py1SA ligand. (b) Excitation (@525 nm emission) and emission (@350 nm excitation) spectra. (c) TC SPC traces of 1 μM Py1SA with and without TTR. (d) Plot of the difference in the fluorescence intensity as a function of the concentration of Py1SA in the presence of a 1 μM solution of TTR in PBS buffer. The Py1SA/TTR ratios 0.5:1, 1:1, 2:1, and 4:1 are indicated by stars. (e) TC SPC—Changes in the decay traces of TTR (1 μM) tryptophans for different concentrations of Py1SA.

Differences in the photophysical properties of the fluorescent Py1SA ligand^19^ [see Figure 1a] in buffer and with TTR can provide valuable evidence for ligand binding. The excitation and emission spectra in PBS and with a 1:1 stochiometric amount of TTR are shown in Figure 1b: We observe a distinct red shift for the excitation and a concomitant blue shift of the emission, which indicates that the Py1SA ligand associates with the hydrophobic binding site. The corresponding time-correlated single photon counting (TC SPC) decay traces at excitation 337 nm were collected at emission in the 520–540 nm region, as shown in Figure 1c. There is a fast single exponential decay in PBS with a decay time of 0.62 (±0.008) ns that becomes considerably elongated upon adding equimolar amounts of TTR. In the latter case, a two-component model can be used to fit the decay. Keeping the first component fixed at 0.62 ns, a slower component of 1.97 (±0.023) ns contributes with 80% amplitude weight. This indicates that not all of the Py1SA are associated with the binding site; however, the elongated decay time upon binding is also consistent with a considerable increase of the fluorescence quantum efficiency from 6.9 (±0.4) to 12.7 (±1.7)% (see Figure S-3 in the SI). Furthermore, a fluorescence binding assay was set up giving a binding curve shown in Figure 1d that could be fitted to a kD of approx. 1.5 μM, being in rough agreement with the time-decay analysis. Another indicator of binding within TTR is to monitor the effect on the fluorescence of tryptophan moieties in terms of fluorescence resonance energy transfer. By stepwise addition of Py1SA to a 1 μM solution of TTR, there is a clear shortening of the tryptophan decay time as shown in Figure 1e. The decay traces go from being single exponential in PBS to a more complicated decay with Py1SA present and was modeled with a double decay. It consists of a slow component associated with only TTR in solution (fixed at: 3.42 ± 0.011 ns) and a much faster component (fitted: 0.561 ± 0.034 ns) gradually growing with increased Py1SA/TTR ratio. At a 4:1 molar ratio of Py1SA/TTR, the latter contributed with 14% amplitude weight. Taken together, the spectroscopic data suggest that Py1SA binds specifically and efficiently to the native protein also at these low concentrations.

### X-ray Diffraction

**Figure 2:**
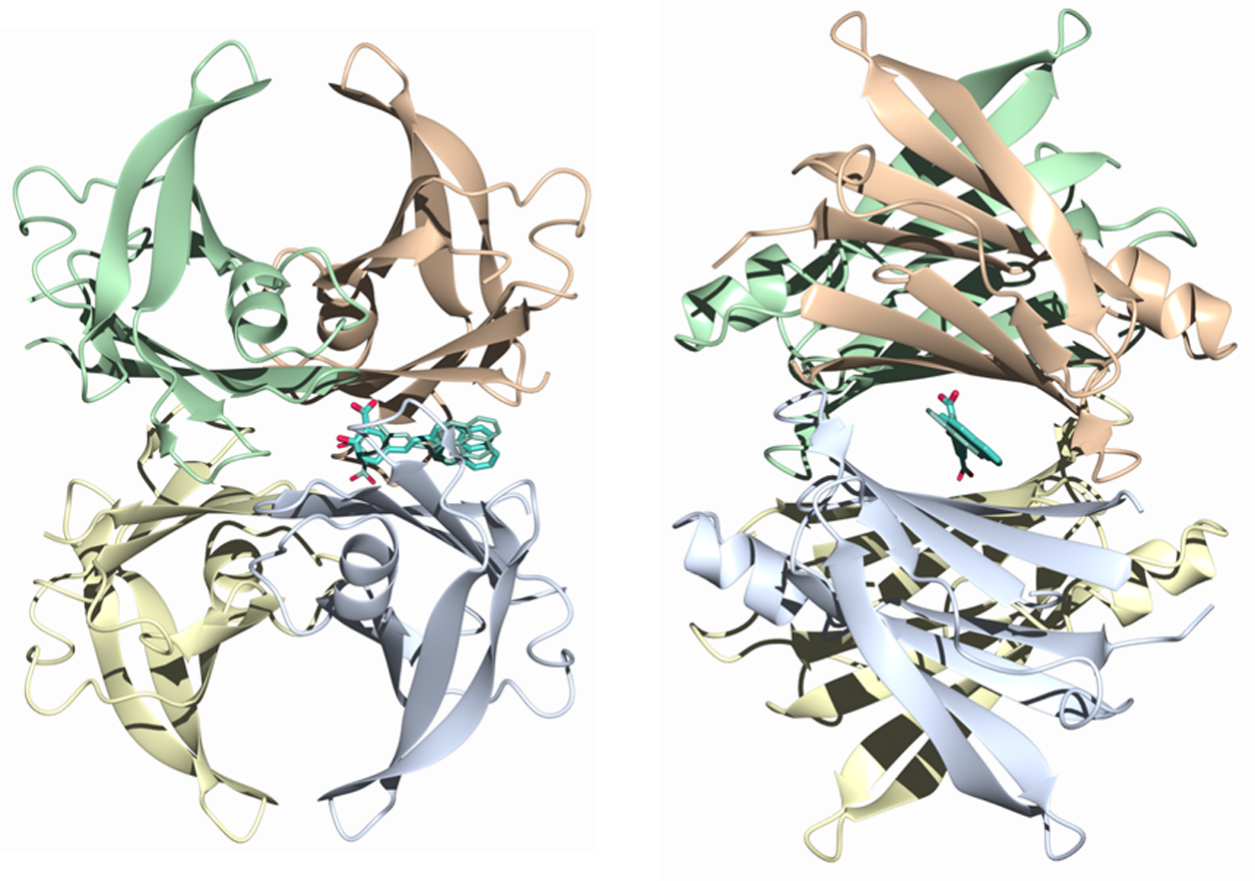
Orthogonal views of the crystal structures of the TTR–Py1SA complex. The individual molecules of the TTR tetramer (shown as ribbons) are highlighted in colors (yellow: A, green: A′, lilac: B, peach: B′). Py1SA (in sticks, here reverse binding mode) binds in the T4 binding site, formed at the B/B′ interface. The equivalent A/A′ site is devoid of ligand.

To gain insight into the ligand binding on the atomistic level, the crystallized TTR–Py1SA complex was investigated by X-ray diffraction. The crystals belong to the space group P2_1_22_1_ with two molecules (the AB dimer) in the asymmetric unit; the second biological dimer (A′B′) forming the tetramer can be obtained by rotation along the crystallographic 2-fold *c*-axis.^17^ The inner β-sheets of the dimer-dimer (AB-A′B′) interface form two ligand-binding site cavities referred to as sites AA′ and BB′, respectively (see Figure 2). These two binding sites are symmetry-equivalent.

**Figure 3:**
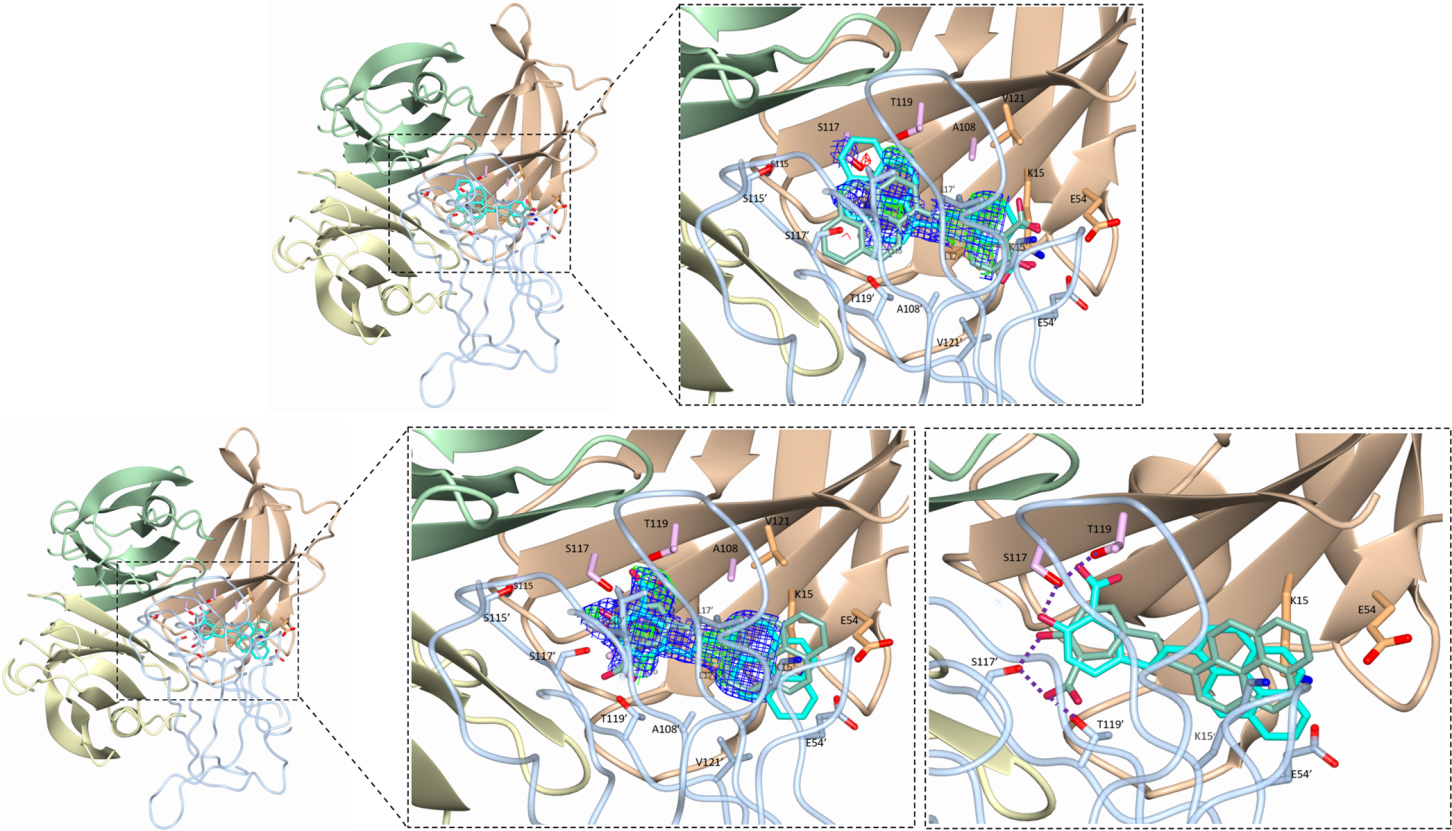
Close-up of TTR–Py1SA binding site, comparing forward (top) and reverse (bottom) binding modes. Forward is defined as when the pyrene moiety is encaged predominantly by HBP 1 (highlighted in pink), while in the reverse case, this moiety is directed along HBP 3 (orange) toward the solvent. In the reverse binding mode, the salicylic group formed hydrogen bonds with Ser-117 and Thr-119. Molecule B′ is depicted in “worm” representation with color-matched side chain “sticks” for clarity. Ligand is represented as sticks with the associated sigma-A weighted maps drawn as a mesh (2mFo-DFc in blue contoured at 1.0 σ and mFo-DFc in green/red contoured at 3.0 σ).

Clear electron density, deviating from solvent (apo TTR), confirmed the presence of the ligand Py1SA as depicted in Figure 3. For model building, ligand “atoms” were “placed” with an occupancy of 0.3 if visible in the electron density, all remaining atoms were “placed” at 0.1 occupancy. Occupancy was increased in line with (i) developing density and (ii) consistency with surrounding B-factors. However, ambiguity in the initial electron density, and lack of any subsequent improvement during the refinement process, did not permit ligand placement with any degree of confidence.

### Molecular Dynamics Simulations

Due to the ambiguity in the interpretation of the X-ray data, we additionally performed molecular dynamics (MD) simulations. The starting points for the MD simulations are the four different possible structures of the TTR–Py1SA complex obtained from the refinement of the X-ray data labeled forward-B, forward-B′, reverse-B, and reverse-B′. Note that forward-B and forward-B′ as well as reverse-B and reverse-B′ represent slightly different structures of symmetry-equivalent binding modes of the TTR–Py1SA complex. These different structures are a result of the electron density being averaged over the AA′ BB′ symmetry axis. In the MD simulations, all protein atoms except for the ones that are within 4 Å from the ligands were under harmonic constraints.^41^

The subsequent 1 μs MD simulations suggest that the reverse mode is more stable than the forward mode. This is supported by several observations: In one of the forward trajectories, the Py1SA ligand tends to move somewhat out of the pocket. In all other trajectories, the ligand is more stationary in the pocket. The unbinding tendency in this trajectory is, e.g., illustrated by the longer distance of the centers of mass of the dye and the pocket (see Figure S-8 in the SI). This movement is also accompanied by larger fluctuations of the Coulombic interaction of the protein with the ligand during the simulation compared to the other trajectories (Figure S-7 in the SI) as well structural changes of the ligand upon leaving the pocket (Figure S-4 in the SI). Further, the reverse mode can establish hydrogen bonds with the pocket (c.f. Figure 3). It shows an average of 3–5 hydrogen bonds (Figure S-6 in the SI). In particular, hydrogen bonds are formed to the residues Ser-117 and Thr-119. This is in line with previous studies, which show that both residues are capable of forming hydrogen bonds with the natural ligand, T4, to stabilize the TTR–T4 complex.^3,42^

We have further investigated the motion of the ligand in the binding pocket during the simulation time. In particular, we find no rotation of the entire ligand, while located in the binding pocket (Figure S-4 in the SI). For the two dihedral angles [ϕ_1_ and ϕ_2_ indicated in Figure 1a], we find simultaneous rather than individual switches within the binding pocket. This can be rationalized by a similar molecular shape and rather small movements necessary for this simultaneous switch compared to rotating about only ϕ_1_ or ϕ_2_. However, the simultaneous switch seems to be a rather rare event (Figure S-5 in the SI).

The Lennard-Jones short-range (LJ-SR), Coulombic short-range (Coul-SR) potential, and their sum are listed in Figure S-7 in the SI. Due to the binding and unbinding process, large fluctuations of the forward-B trajectory are observed in particular in the Coulomb contribution. The overall short-range binding energy for the other forward trajectory is higher than that for the reverse trajectories by about 160–170 kJ/mol. The shifts observed in the optical spectra suggest a hydrophobic character of the binding pocket. This observation is in line with the identified pocket, as it contains apart from Ser-117 and Thr-119 at the inner binding pocket (which establish hydrogen bonds to the ligand) mainly amino acids of hydrophobic nature.

### Umbrella Sampling Simulations

This difference in the binding energy, however, is not conclusive of the actual free binding energies. For this purpose, we additionally performed umbrella sampling (US) simulations to construct the potential mean force (PMF) surface starting from all four initial structures of the TTR–Py1SA complex. In the pulling trajectories, we increased the distance of the center of the TTR binding pocket to the Py1SA molecule.

**Figure 4:**
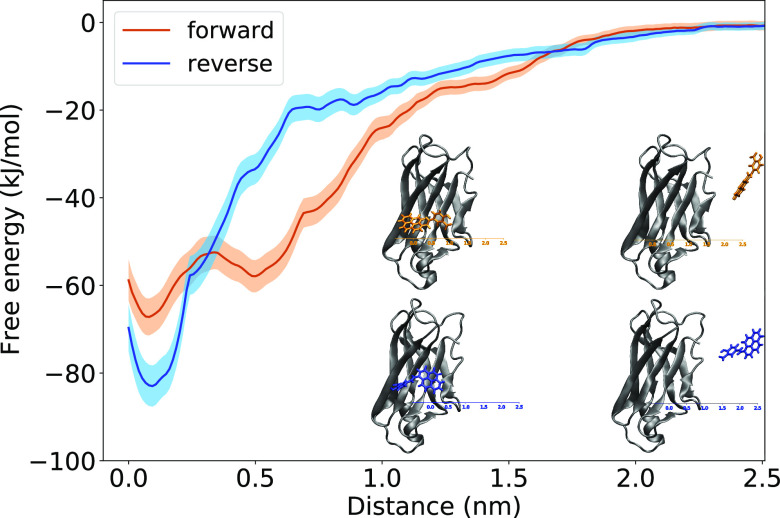
Free energy profiles for Py1SA in the forward (orange) and reverse (blue) modes obtained by the potential of mean force approach by pulling the ligand from the binding site to become free in solution.

The corresponding data for all calculated pathways can be found in Figures S-9 to S-12 in the SI. We have selected the trajectories for the potential of mean force analysis and binding free energy calculation as outlined in Section S-4 in the SI. The averaged PMF profiles for the forward and reverse modes obtained by evaluating the distance histograms of the simulations by employing the WHAM algorithm are shown in Figure 4.

For both binding modes, we observe the deepest minimum within the first 0.5 nm from the starting point. The binding free energy was calculated by taking the difference between the last and the lowest values of the PMF graphs. We observe binding free energies of 67 ± 4 and 83 ± 5 kJ/mol for the forward and reverse modes, respectively. This corresponds to a clear domination of the reverse mode. This finding is in line with the MD results discussed above and a previous study where the structure–activity relationships^4^ revealed the importance of the presence of the carboxylic acid as well as its position in the ligand structure in its activity.

## Summary and Conclusions

There is an unmet need in the field of amyloidosis to easily and accurately diagnose and monitor patients during treatment with different treatment modalities. TTR in TTR amyloidosis is a particularly interesting target due to its abundance of accessible blood samples and numerous successful available treatments. While this protein and its associated diseases have been researched for a long time, there is currently no clinically approved TTR-based biomarker for TTR amyloidosis in blood plasma or cerebrospinal fluid. One considerable issue is distinguishing misfolded TTR from native tetrameric TTR. Our work herein and previously on fluorescent amyloid ligands that can distinguish misfolded fibrillar TTR and native TTR is a development toward that end.^20^ Furthermore, detailed knowledge of the binding modes of various small-molecule ligands toward the native TTR tetramer can lead to new kinetic stabilizers as alternatives to diflunisal and tafamidis currently approved as anti-TTR amyloid drugs.^43^ The native TTR tetramer with its intrinsic symmetry poses a challenge for X-ray crystallography due to the partial occupancy necessarily observed due to the crystal lattice. In this work, we have therefore complemented our biophysical and structural work with molecular dynamics simulations to establish the most plausible ligand–TTR complex structure at atomic resolution of TTR–Py1SA. The work described in this study represents a successful methodology for mitigating the issue of partial ligand occupancy.

## Supporting Information Available

Experimental and computational details (Section S-1); interim refinement statistics for the analysis of the X-ray diffraction data (Section S-1.4); absorption and emission spectra as well as quantum efficiency plots for Py1SA in PBS in the presence of TTR (Section S-2); detailed analysis of the molecular dynamics simulations (Section S-3); detailed analysis of the umbrella sampling simulations (Section S-4); additional data for the computational part of the study including pdb files of the simulated structures of the two binding modes after the equilibration step can be found in ref (44) (PDF)
