# Supplementary material for: Binding of a Pyrene-Based Fluorescent Amyloid Ligand to Transthyretin: A Combined Crystallographic and Molecular Dynamics Study
Source: J Phys Chem B. 2023 Jul 21;127(30):6628–35. doi: 10.1021/acs.jpcb.3c02147 (PMC10405211; doi:10.1021/acs.jpcb.3c02147)
Supplement: Supplementary file 1 — jp3c02147_si_001.pdf [file jp3c02147_si_001.pdf]

Supporting Information to Binding of pyrene-based fluorescent amyloid ligand to transthyretin: A combined crystallographic and molecular dynamics study

Nghia Nguyen Thi Minh,[†] Afshan Begum,[‡] Jun Zhang,[‡] Petter Leira,[¶] Yogesh Todarwal,[§] Mathieu Linares,[§,‖,⊥] Patrick Norman,[§] Dean Derbyshire,[‡] Eleonore von Castelmur,[‡] Mikael Lindgren,[¶] Per Hammarström,[*,‡] and Carolin König[*,†]

† *Institute of Physical Chemistry and Electrochemistry, Leibniz University Hannover, Callinstr. 3A, 30167 Hannover, Germany.*

‡ *Division of Chemistry Department of Physics, Chemistry and Biology, Linköping University, 581 83, Linköping, Sweden*

¶ *Department of Physics, Norwegian University of Science and Technology, 7491 Trondheim, Norway*

§ *Department of Theoretical Chemistry and Biology, School of Engineering Sciences in Chemistry, Biotechnology and Health, KTH Royal Institute of Technology, SE-106 91 Stockholm, Sweden*

‖ *Laboratory of Organic Electronics, ITN, Linköping University, SE-581 83 Linköping, Sweden*

⊥ *Scientific Visualization Group, ITN, Linköping University, SE-581 83, Linköping, Sweden*

E-mail: per.hammarstrom@liu.se; carolin.koenig@pci.uni-hannover.de

S-1 Methodology

S-1.1 Fluorescence measurements of Py1SA ligand binding to TTR in solution

The photophysical properties in phosphate-buffered saline (PBS) buffer and with TTR present were examined using steady-state and time-resolved fluorescence. For the former, excitation and emission spectra were collected using typically 1 µM TTR and Py1SA in buffer employing a PTI Quantamaster 8075-22 (Horiba Scientific) equipped with Double Mono 300 spectrometer chambers. Time-resolved fluorescence decays were recorded using an IBH time-correlated single photon counting (TC SPC) spectrometer system using an ns LED operating at 337 nm. For details on experimental procedures and signal processing see refs. 1,2; see caption to Figure 1 in the main text and S-3 for detailed settings. To see how the emission changed upon the binding between the Py1SA ligand and TTR, titrations to approximate the dissociation constant (Kd) were performed with increasing concentrations of Py1SA to 0.5 µM TTR tetramer using a fluorescence plate reader (Tecan Infinity M1000). Fitting was performed to the standard hyperbolic function using OriginPro.

S-1.2 Recombinant expression and purification of TTR

Expression and purification of human TTR were carried out as described previously.[3] Competent *Escherichia coli* BL21 (DE3) cells were transformed with TTR-gene containing pET-3a plasmids and were grown overnight on LB agar plates containing 100 µg/mL ampicillin. A few single colonies were transferred to LB media supplemented with 100 µg/mL ampicillin and grown with orbital shaking at 37 °C. The cells were grown until an OD_{600} of 0.4 then the temperature was lowered to 20 °C and the cells were grown for another 30 min (to an OD_{600} of 0.6) followed by induction with 0.4 mM isopropyl thiogalactopyranoside (IPTG) at 20 °C.

After 18 hours of protein expression, the cell pellet was harvested by centrifugation

and was resuspended in 20 mM Tris-HCl, pH 8.0, 100 mM NaCl, (Buffer A) and lysed by sonication. The lysate was cleared by centrifugation at 25,000 × g for 30 min at 4 °C. The supernatant was heated to 60 °C for 30 min. After heating, the precipitated material was removed by centrifugation at 14,000 × g for 30 min at 4 °C followed by filtration through 0.45 µm cellulose acetate membrane (Millipore) and applied to a Source-15Q 10/10 ion exchange chromatography column. The column was washed extensively with buffer A and elution was carried out with a linear gradient of 1M NaCl in buffer A. Sample containing TTR was further purified by a subsequent size-exclusion chromatography on a HiPrep 16/60 superdex 75 column (Cytiva) equilibrated with 10 mM Na-phosphate buffer, 100 mM KCl pH 7.6 at 20 °C. Fractions containing pure TTR were collected, pooled, and concentrated using an Amicon Ultra centrifugal filter device (Millipore, 3 kDa molecular-weight cutoff). Protein concentration was determined by using the absorption extinction coefficient 73,156 $M^{-1}cm^{-1}$ at 280 nm applied for tetrameric TTR. Protein quality and purity were accessed by SDS-PAGE prior to experiments. Aliquots of purified TTR were flash-cooled in liquid nitrogen and stored at -80 °C until use.

S-1.3 Crystallization of the Py1SA–TTR complex

The protein was crystallized as described previously.[3] The purified TTR was dialyzed against 10 mM Na-phosphate buffer with 100 mM KCl (pH 7.6) and concentrated to 5.2 $mg{\cdot}mL^{-1}$ using an Amicon Ultra centrifugal filter device (Millipore, 3 kDa molecular-weight cutoff) and co-crystallized at room temperature with 500 µM concentration of Py1SA added from DMSO stock solutions at 10 mM, using the vapor-diffusion hanging drop method. A drop containing 3 µL protein solution was mixed with 3 µL precipitant and equilibrated against 1 mL reservoir solution containing 1.3–1.6 M sodium citrate and 3.5 % v/v glycerol at pH 5.5 in 24-well Linbro-plates. Crystals grew to dimensions of 0.1 × 0.1 × 0.4 mm^3 after 5-7 days. Once fully grown, the crystals were further transferred into a new equilibrated drop containing the same amount of ligand and were incubated for three days. The crystals were

cryo-protected with 12.5 % v/v glycerol and to avoid the possibility of the ligand washing out of the crystals during the brief cryo-protection step, the final cryo-solution always contained the same amount of ligand.

S-1.4 X-ray data collection, integration, and processing

The X-ray diffraction data of Py1SA–TTR were collected under cryogenic conditions at the MAX IV facility (MAXIV), Sweden, using PILATUS detectors at a wavelength of 0.97993 Å. These data were processed to a resolution of 1.4 Å using XDS[4]and AIMLESS from the CCP4 software suite.[5] Data collection statistics are summarized in Table S-I. Phasing was done by molecular replacement using Phaser[6] with a search model derived from the published coordinates 1F41. In short, residues 11–98 and 104–122 were included in the initial model omitting a known flexible region. The model was refined against all the diffraction data using REFMAC.[7] Manual map inspections were performed with COOT.[8] Ligands and solvent were placed in density after 1 to 2 rounds of rebuilding the protein model with COOT and refinement using REFMAC.

Table S-I: Data collection and interim refinement statistics

**Data collection***		
Resolution	35.61 – 1.4 Å	
Space group	$P2_122_1$	
Cell parameters: a, b, c (Å)	43.018, 64.434, 85.441	
α, β, χ (°)	90.00, 90.00, 90.00	
Completeness	98.8 (97.8)	
Redundancy	6.5 (6.8)	
Rmerge	0.059 (1.128)	
Rpim	0.025 (0.464)	
I/sigI	13.3 (1.5)	
CC1/2	0.998 (0.627)	
* Values in parentheses are for the highest-resolution shell		
**Current model** (2 rounds of COOT & REFMAC post ligand placement)	TTR + Py1SA (forward)	TTR + Py1SA (reverse)
Protein	2 chains: 116, 114 residues	2 chains: 116, 114 residues
Waters	158 waters	158 waters
	1 Py1SA molecule	1 Py1SA molecule
R factor	0.1453	0.1447
'free' R factor	0.1865	0.1847
Real-space correlation coefficient	0.966	0.956
G values:		
Dihedrals	-0.18	-0.19
Covalent	0.31	0.31
Overall	0.03	0.02
Estimated coordinate error (DPI)	0.0544	0.0539
Ligand validation (individual):		
Real-space R factor	- / 0.246	- / 0.172
Real-space correlation coefficient	- / 0.766	- / 0.888

S-1.5 Forcefield parameters for Py1SA

The geometry optimization using the Gaussian (version 16.B.01) program[9] was initially performed at the B3LYP level of theory in combination with the 6-31G(d,p) basis set to identify the lowest energy conformers. Next, the initial forcefield was generated by deriving the RESP charges for the most stable conformer (Conf3 in Fig. S-1) with B3LYP/6-31G* (as recommended for RESP charges[10]) and incorporating the remaining parameters from the General Amber Force Field (GAFF).[11,12] The equilibrium bond distance and bond angle parameters were further improved based on an optimized structure (B3LYP/6-31G(d,p)). The dihedral potentials of ϕ_1 and ϕ_2 (marked in Fig. S-1) calculated from Molecular Mechanics (MM) were fitted to the DFT potential (see Fig. S-2). For a detailed procedure about force-field parametrization, refer to supporting information of our previous work.[13]

To validate the forcefield, the ground state energies of conformers computed using MM and DFT methods were compared (see Table S-II). The highest error between the two meth-

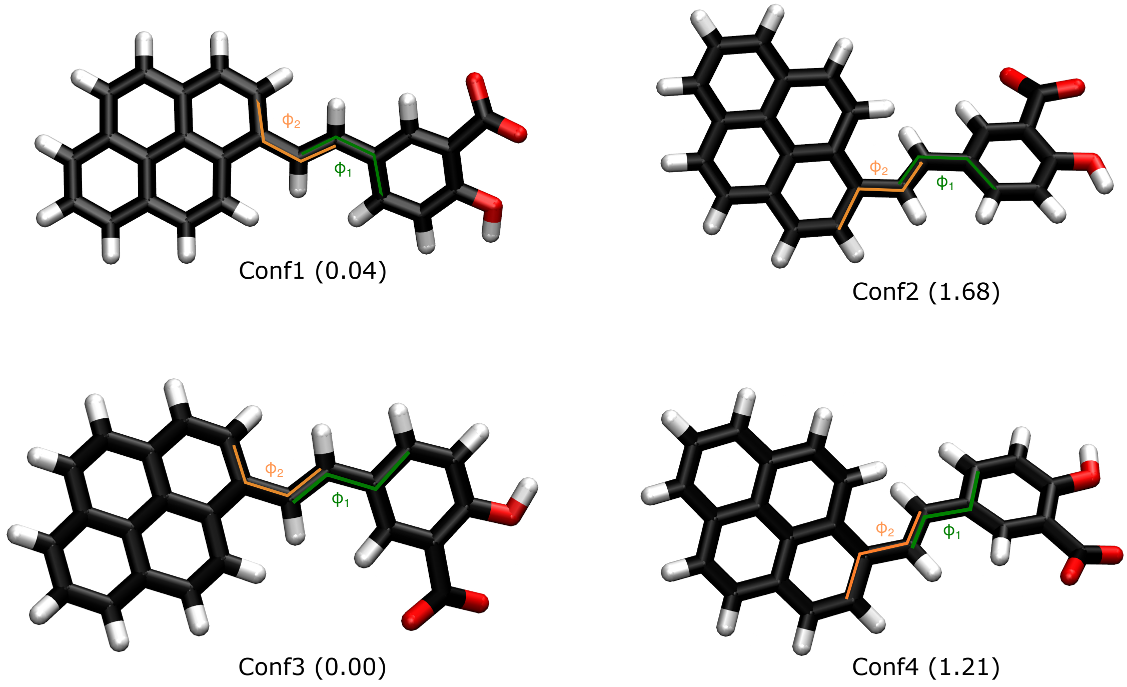

Figure S-1: Optimized molecular structure of Py1SA conformations with labels indicating important dihedrals and relative energy in kcal/mol with respect to the most stable conformer (B3LYP/6-31G(d,p)).

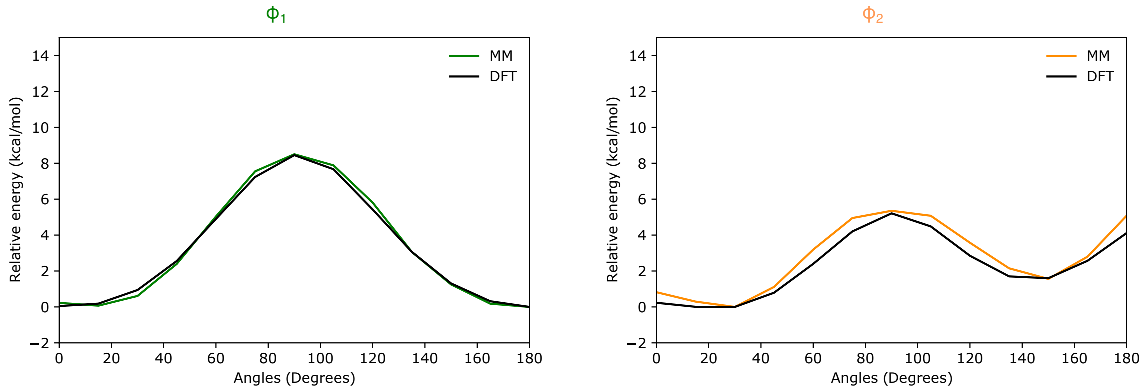

Figure S-2: Energy profiles for relaxed scans varying the dihedral angles ϕ_1 (left) and ϕ_2 (right) of Py1SA marked in Fig. S-1 with the fitted force field (MM) and B3LYP/6-31G(d,p) (DFT), respectively.

Table S-II: Comparison of DFT vs. MM relative ground-state energy of Py1SA conformers optimized with the respective method.

	conf1	conf2	conf3	conf4
MM Relative Energy(kcal/mol)	-0.31	1.70	0.00	1.64
DFT Relative Energy(kcal/mol)	0.04	1.68	0.00	1.21
Error (kcal/mol)	0.35	0.02	0.00	0.43

ods was found to be 0.43 kcal/mol for conf4, which falls within the error range of the DFT method.

S-1.6 Molecular dynamics simulations

All molecular dynamics (MD) simulations were performed using the Gromacs version 2019.3[14] with the Amber ff14SB force field[15] for the TTR protein and re-parametrized General Amber Force Field (GAFF)[11,12] for Py1SA (see Section S-1.5).

The starting structures of the TTR protein with Py1SA ligand existing in two configurations with respect to protein were based on the X-ray crystallography data from the present study (see Figure 3 in the main text). As a result of electron density being averaged along the AA' BB' symmetry axis, crystallography could not differentiate between four potential binding models for the ligand, i.e., forward-B, forward-B', reverse-B, and reverse-B'. All models were presented as possible starting points for modeling. All initial structures from

the X-ray analysis were cleaned using the module pdbfixer,[16] Gromacs[14] utilities, and the Gauss view[17] program by removing water molecules, adding H atoms to the protein and the ligand, and adding three missing amino acid residues to the two chains of the TTR protein.

We used Gromacs tools to solvate the protein–ligand system in a TIP3P[18] water box of size $7 \times 7 \times 9$ nm^3 with a total of approximately 12,000 water molecules. 21 Na^+ ions were then added to obtain system charge neutrality. The long-range electrostatic interactions were calculated using the particle mesh Ewald (PME) method[19] with a long-range cutoff of 1 nm which also is the cutoff of the short-range van der Waals interactions. Default settings were used for Fourier spacing (0.12 nm) and PME order (4). Periodic boundary conditions were applied to all three directions of the simulation box. The energy minimization was then performed using the steepest decent algorithm with a maximum step size of 0.001 nm and a maximum force of 10 kJ mol^{-1}nm^{-1}. Subsequently, all protein atoms except the ones that are within 4 Å from the ligand (4 Å pocket) were under the harmonic constraints with the strength of 1,000 kJ mol^{-1}nm^{-2}. In addition, the LINCS algorithm[20] was used to constrain all bonds. We first performed short 100 ps equilibration in the NVT 300 K ensemble using velocity rescaling, followed by a 100 ps equilibration in the NPT ensemble at 1 atm and 300 K. The coupling time used is 0.2 ps for the temperature and 1.0 ps for the pressure. Subsequently, the MD simulations were performed in the NVT ensemble for 1 µs simulation time. Processing of the different raw data from MD production was done using Gromacs utilities. Hydrogen bond analysis was performed with the *gmx hbond* program using the default definition of H-bond, i.e., the angle H–donor–acceptor should be $\leq 30°$ and the distance donor—acceptor ≤ 0.35 nm.

S-1.7 Umbrella sampling simulations

We computed the potential of mean force (PMF) surface using umbrella sampling.[21–23] MD simulations were employed to extract the initial coordinates for binding free energy calculations. We applied the same computational settings as in the molecular dynamics simulations

but using Gromacs version 2021.3[14] and except the box size was set to $7 \times 11 \times 9$ nm^3. We further only constrained the backbone throughout the protein in the US simulations. Starting from the last snapshot of each initial structure from the equilibration phase, Py1SA was pulled away from its binding site about 2.5 nm; we used a force of 5,000 kJ mol^{-1}nm^{-2} for the first (B) and 8,000 kJ mol^{-1}nm^{-2} for the second structure (B') of reverse mode, 7,000 kJ mol^{-1}nm^{-2} for the first (B) and 8,000 kJ mol^{-1}nm^{-2} for the other structure (B') of forward mode. The Py1SA was pulled at the rate of 0.005 nm/ps. The spring constant as well as the pulling rate in each simulation were carefully selected based on our empirical study so that large overlaps of the histograms were obtained (see Section S-4). From these pulling trajectories, snapshots with equivalent distance of 0.025 nm were set as a starting configuration for each umbrella sampling simulation, which was independently simulated by performing an NPT equilibration for 100 ps followed by a 1 ns NVT trajectory. The force constant of the umbrella potential is set to 4,000 kJ mol^{-1}nm^{-2} for all trajectories. Each simulation consists of a total of 101 umbrella windows. Additionally, we added 17 extra windows between 0.2 nm and 0.4 nm for each trajectory of these modes. Thus in total, for these modes, each simulation consists of 118 windows.

Finally, the weighted histogram analysis method (WHAM)[24,25] was used to combine several windows into a PMF curve to estimate the binding free energy. For each initial structure, two pulling simulations followed by umbrella sampling were conducted to get the average PMF curve.

Molecular graphics were prepared using the VMD program.[26]

S-2 Spectroscopic evidence for Py1SA ligand binding to TTR in solution

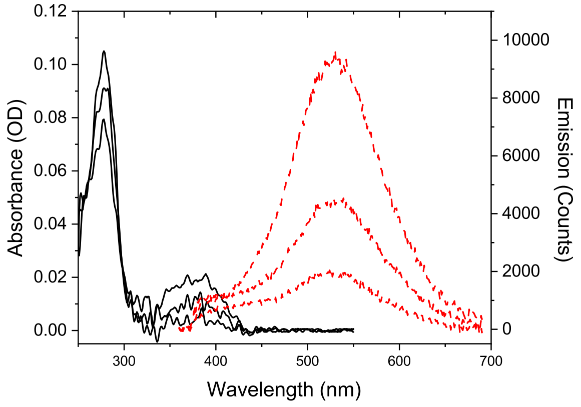

(a)

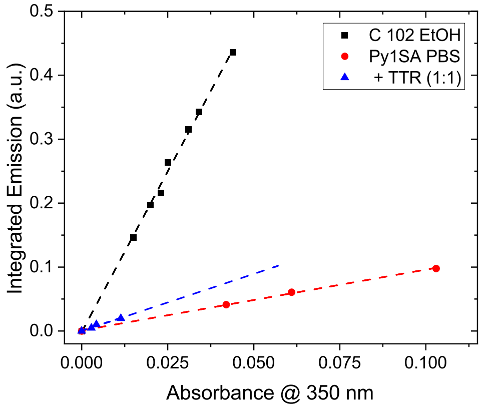

(b)

Figure S-3: Absorption and emission spectra and quantum efficiency plots of the Py1SA fluorescent ligand in PBS in the presence of TTR. a) Representative absorption (black) and emission (red) spectra for the Py1SA:TTR system. b) Plots of total emission vs. absorption giving the quantum efficiency from the slope values. Here also data of Py1SA in PBS and the quantum efficiency standard Coumarin 102 (C102) in ethanol, are shown.[27]

S-3 Molecular dynamics simulations

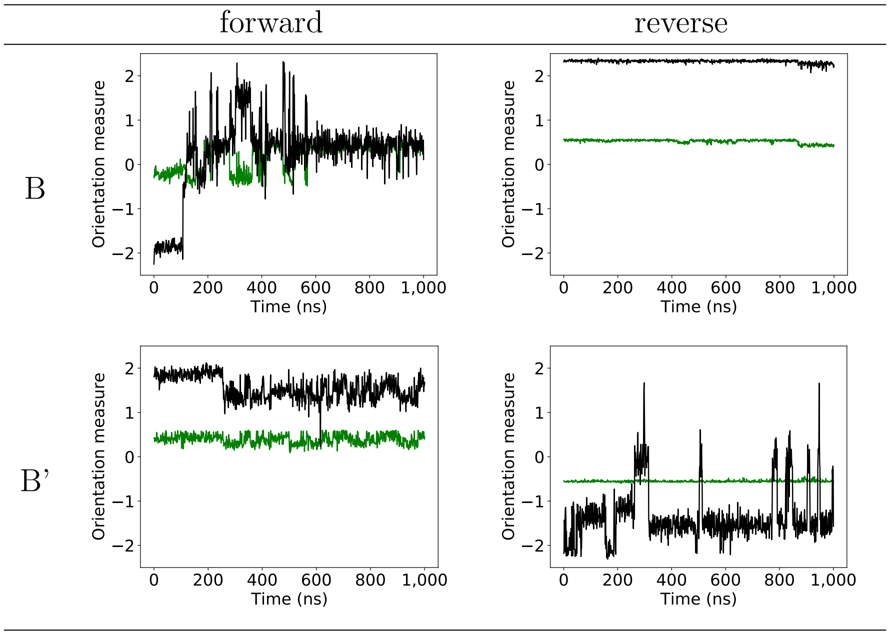

Figure S-4: Projection of vector 1 (v_1, green, showing the orientation of the salicylic acid group) and vector 2 (v_2, black, showing the orientation of the pyrene group) on the respective reference vectors of reverse-B conformer during the simulation time as a measure of orientation. For the definition of v_1 and v_2 see Figure 1 (a) in the main text. In none of the trajectories, we observe a sign change in the orientation of both, the salicylic acid group and the pyrene group. That means we observe no rotation of the ligand inside the pocket. The relatively large change in orientation measures in the forward-B trajectory is accompanied by an unbinding/binding process. The observed rotations of the pyrene group in the reverse-B' trajectory represent a rotation of this group at the edge of the pocket, while the orientation of the more buried salicylic acid group is very stable in the binding pocket.

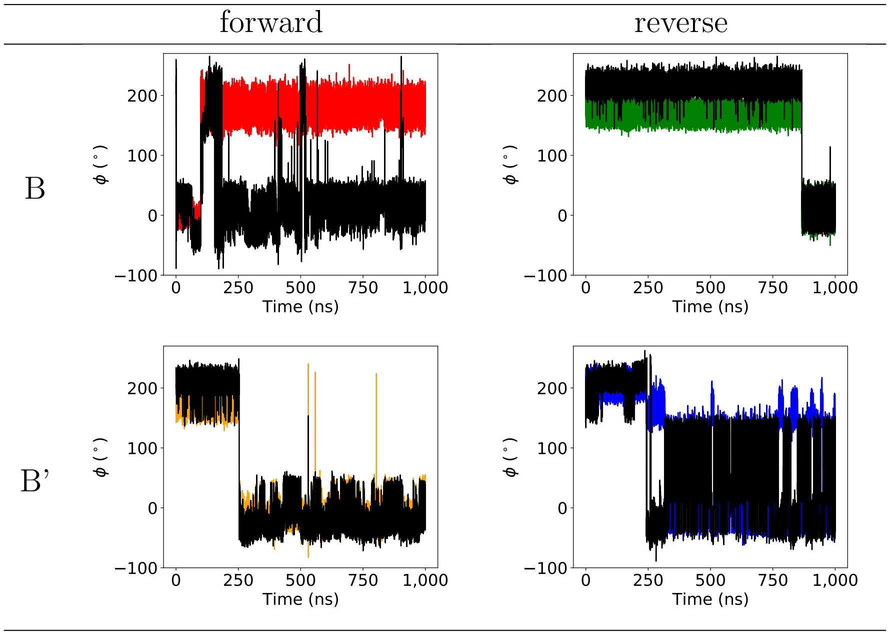

Figure S-5: Dihedral ϕ_1 (color) and ϕ_2 (black) (for the definition of ϕ_1 and ϕ_2 see Figure 1 (a) in the main text) in the Py1SA the simulation time. ϕ_1 and ϕ_2 exhibit few changes over the simulation time. Notably, we observe a tendency to simultaneous switches of both dihedral angles for instance for forward-B’ at around 250 ns and for reverse-B and about 850 ns. This simultaneous switch may be favored over individual switches as the molecular shape remains similar during this procedure.

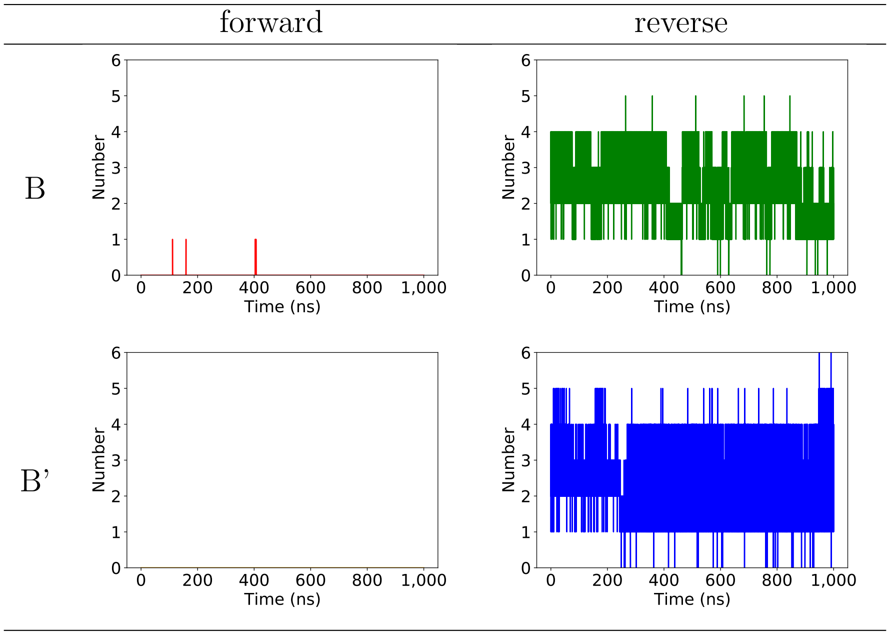

Figure S-6: Number of hydrogen bonds during 1 μs simulation (red for forward-B mode, green for reverse-B mode, orange for forward-B’ mode, and blue for reverse-B’ mode), as defined by the Gromacs utilities. We observe significantly more hydrogen bonds between Py1SA and the 4 Å-pocket for the reverse mode than for the forward mode.

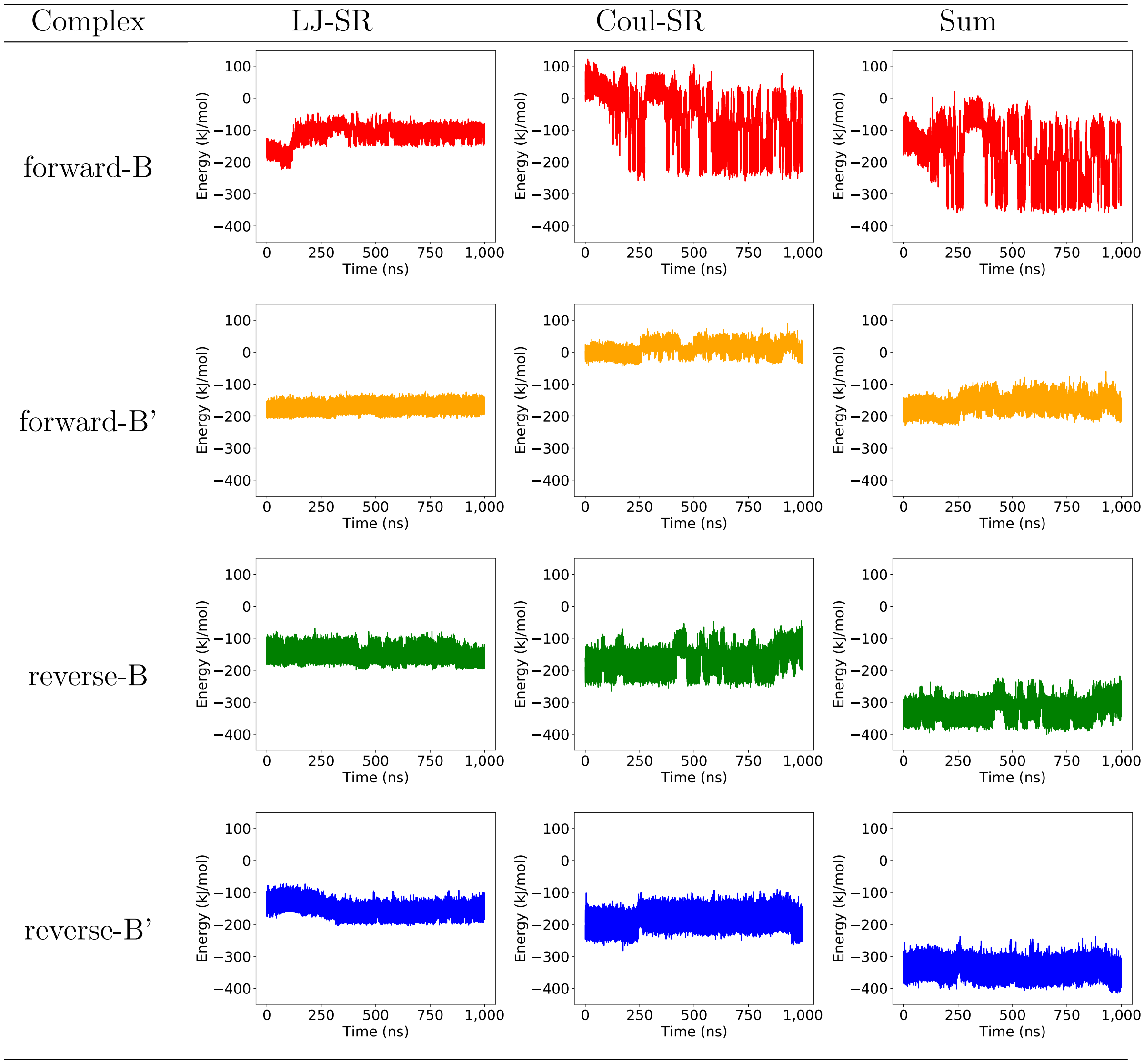

Figure S-7: Short-range interaction (Lennard–Jones short–range (LJ–SR), Coulombic short-range (Coul–SR)) energy between ligand and protein during 1 μs MD simulations. While the average attractive contribution to the interaction energy of LJ-SR in forward modes (-116 kJ/mol for forward-B, -175 kJ/mol for forward-B') is greater than Coul-SR potential (-57 kJ/mol and 10 kJ/mol, respectively) the contribution of these interactions in reverse mode is very similar. In general, the overall interaction energy for the forward mode is higher than that for the reverse one by about 160–170 kJ/mol.

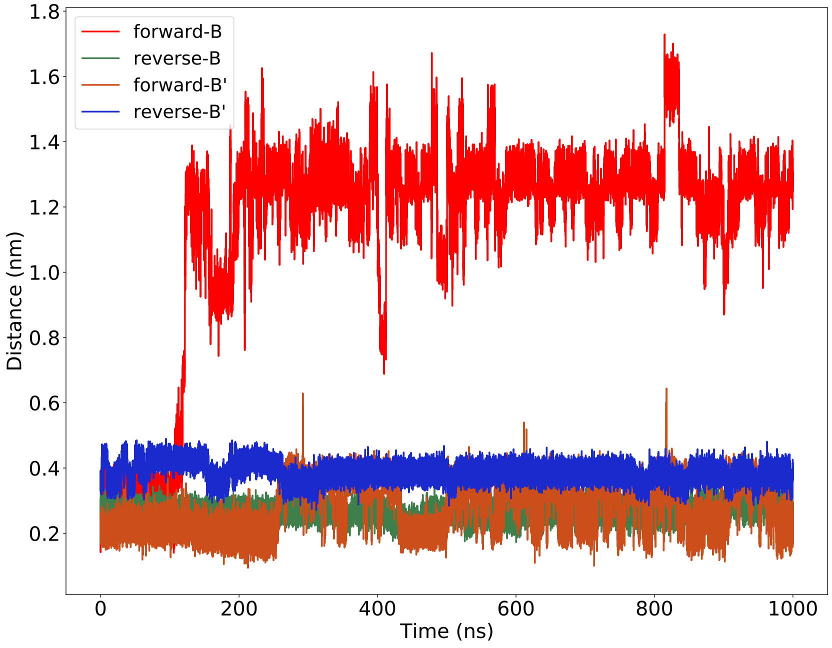

Figure S-8: Distance of the center of mass (COM) of the pocket and the COM of Py1SA along the MD trajectories. In the forward-B mode, there is a noticeable increase in the distance between the pocket's COM and the ligand's COM, which also fluctuates during the MD simulation.

S-4 Umbrella sampling

We have obtained two PMF trajectories for every starting structure. The results are shown in Figures S-9 – S-12. In our analysis, we focus on the results from the forward-B' and reverse-B'. We disregard forward-B due to rather pronounced Coulombic and hydrogen-bond interaction in the pulling trajectories at distances up to more than 1.5 nm from the original binding pocket (see the first trajectory of forward-B in Figure S-10). The second trajectory of forward-B exhibits Lennard–Jones interaction at about the same distance. In both cases, the PMF shows significantly smaller energetic differences between the minimum and endpoint than for the trajectories for the forward-B' (Figure S-9). These observations suggest that the unbinding process in these two trajectories is not fully finished so we cannot conclude on a free binding energy in case of the pulling trajectories obtained from the forward-B initial structure. Similar Coulombic interaction is also obtained for the second pulling trajectory of the reverse-B' initial structure and first pulling trajectory of reverse-B initial structure (see Figure S-12). In contrast to the first case, the latter case is also accompanied by a smaller energetic difference of the minimum with the endpoint. This is why we disregarded this trajectory.

For the reverse-B' trajectories, we observe a shift in the ϕ_1 angle close to the minimum for the pulling trajectories which we do not observe for the remaining pulling trajectory of reverse-B (*c.f.* Figure S-12). However, due to the small number of pulling trajectories, there is no clear evidence for another minor binding mode driven by the ϕ_1 angle. If we include the PMF for reverse-B in the bootstrap for the reverse mode, we obtain a binding free energy of 80±4 kJ/mol and if we do treat speculative possible minor modes separately, we obtain binding free energies of 83±5 and 78±4 kJ/mol for the reverse modes. All reverse mode binding free energies are, hence, clearly larger than the 67±4 kJ/mol obtained for the forward modes. This leaves the conclusion unchanged that the reverse mode is dominant. In the main text, we only show the PMFs obtained from the forward-B' and reverse-B' initial structures.

Traj.	Displacement	Force	Histogram	Profile	Δ (kJ/mol)
1	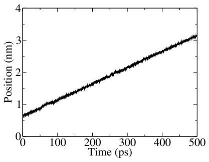	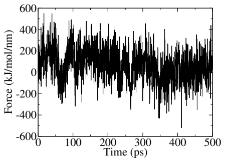	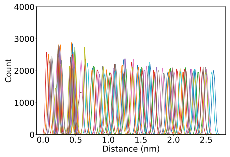	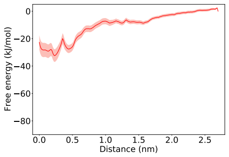	$-29\pm5/-32\pm5$
2	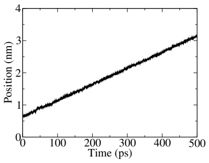	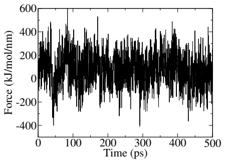	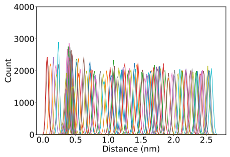	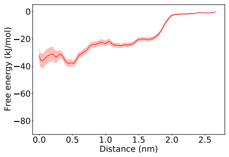	$-36\pm5/-38\pm3$
3	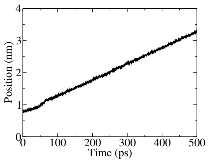	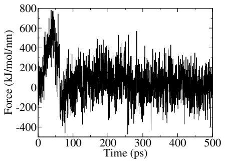	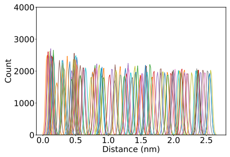	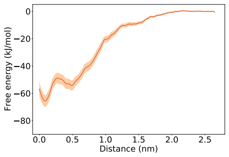	-66 ± 4
4	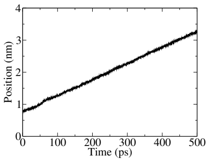	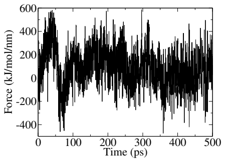	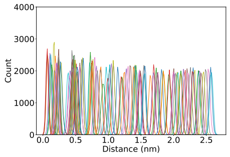	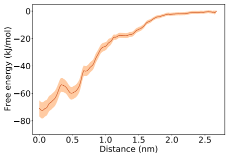	-72 ± 6
3+4			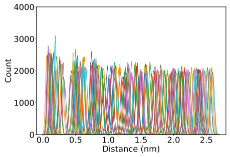	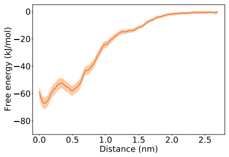	-67 ± 4

Figure S-9: Displacement, force on the spring over time, bootstrap profile, histogram, and binding free energy (Δ, in kJ/mol) of two independent trajectories (Traj.) derived from 118 fully independent sets of umbrella simulations for forward-B (trajectory 1, 2) and B' (trajectory 3, 4) initial structures (pulling force = 4000 kJ mol^{-1} nm^{-2}, pulling rate = 0.005 nm/ps, pulling distance = 2.5 nm).

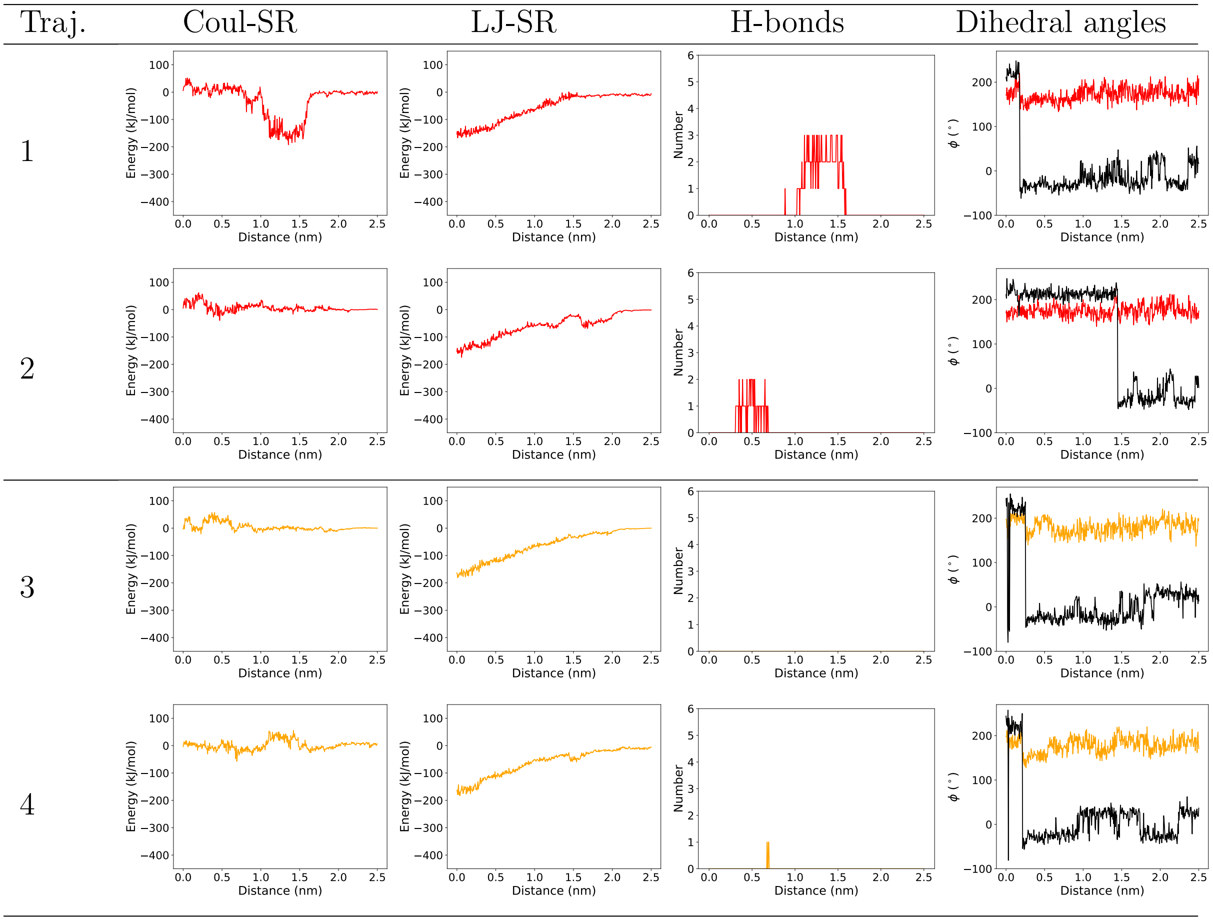

Figure S-10: Coulomb and Lennard–Jones interactions, number of hydrogen bonds between the ligand and protein, dihedral angles ϕ_1 (black) and ϕ_2 (red) of 2 independent pulling trajectories (Traj.) of forward-B (trajectory 1, 2) and B' (trajectory 3, 4) initial structures (pulling force = 4000 kJ mol^{-1} nm^{-2}, pulling rate = 0.005 nm/ps, pulling distance = 2.5 nm).

Traj.	Displacement	Force	Histogram	Profile	Δ (kJ/mol)
1	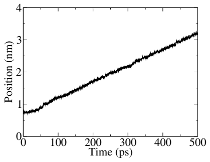	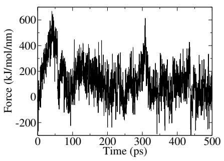	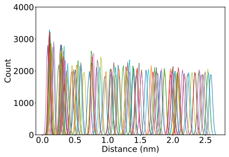	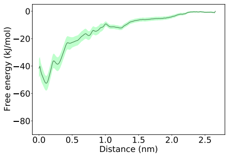	−52±5
2	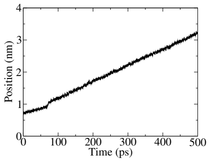	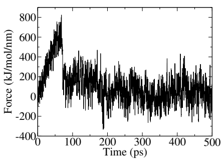	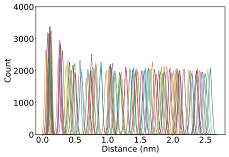	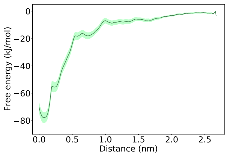	−78±4
3	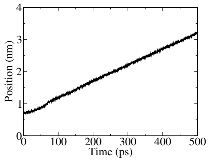	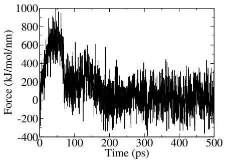	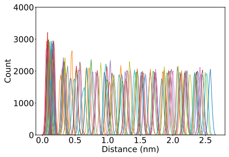	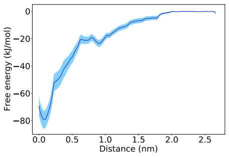	−79±7
4	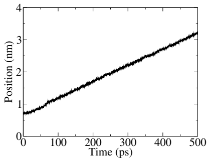	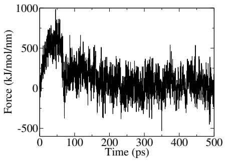	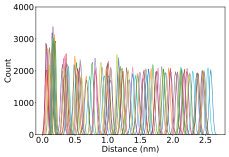	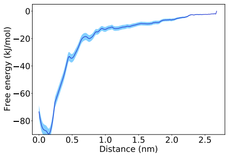	−90±4
3+4			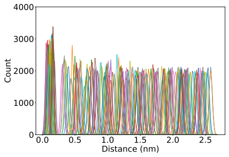	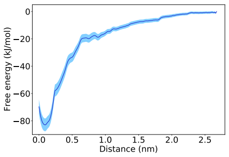	−83±5
2+3+4			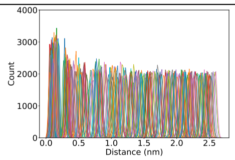	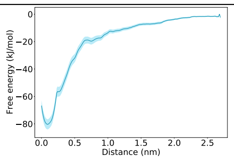	−80±4

Figure S-11: Displacement, force on the spring over time, bootstrap profile, histogram and binding free energy (Δ, in kJ/mol) of two independent trajectories (Traj.) derived from 118 fully independent sets of umbrella simulations for reverse-B (trajectory 1, 2) and B' (trajectory 3, 4) initial structures (pulling force = 4000 kJ mol^{-1} nm^{-2}, pulling rate = 0.005 nm/ps, pulling distance = 2.5 nm).

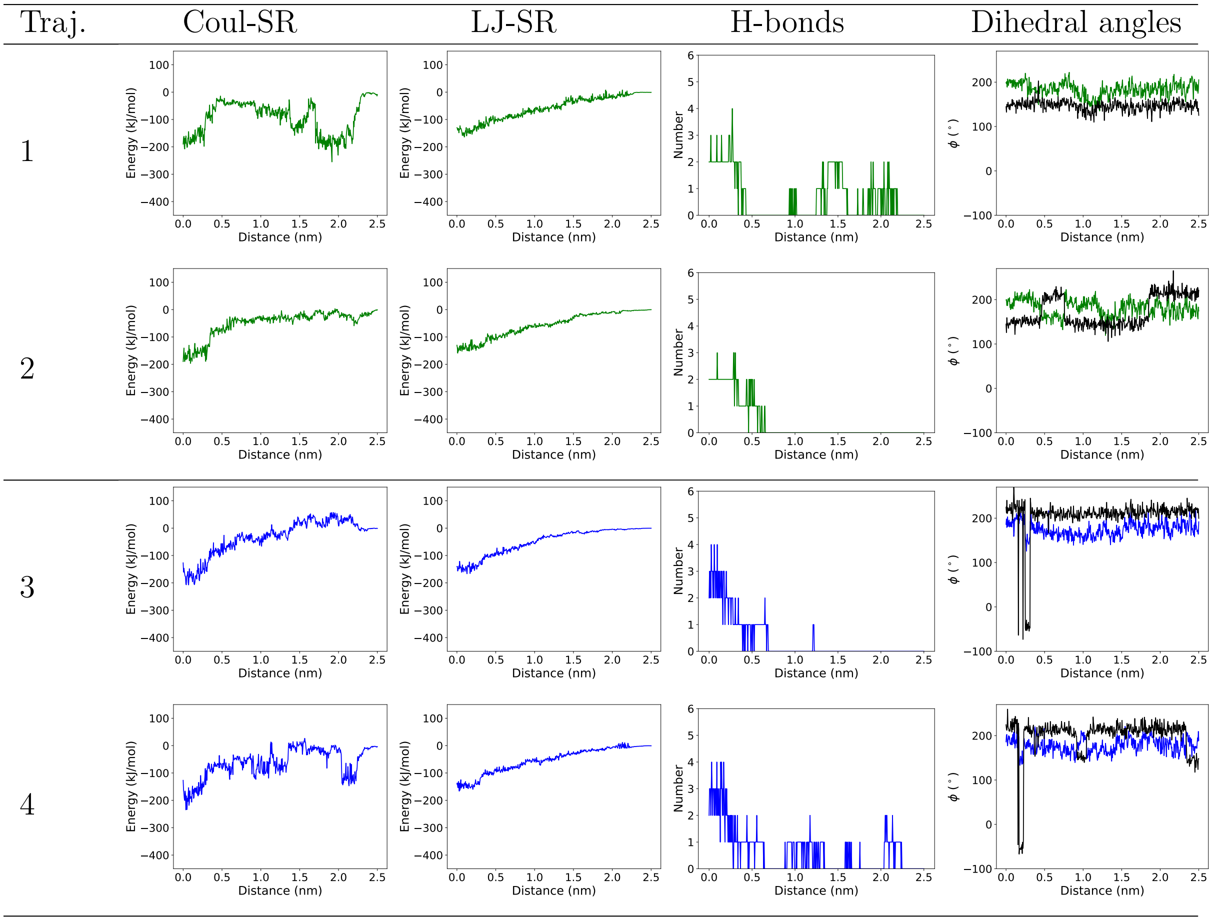

Figure S-12: Coulomb and Lennard–Jones interactions, number of hydrogen bonds between the ligand and protein, dihedral angles ϕ_1 (black) and ϕ_2 (orange) of two independent pulling trajectories (Traj.) of reverse-B (trajectory 1, 2) and B' (trajectory 3, 4) intial structures (pulling force = 4000 kJ mol^{-1} nm^{-2}, pulling rate = 0.005 nm/ps, pulling distance = 2.5 nm).

References

(1) Gustafsson, C.; Shirani, H.; Leira, P.; Rehn, D. R.; Linares, M.; Nilsson, K. P. R.; Norman, P.; Lindgren, M. Deciphering the electronic transitions of thiophene-based donor-acceptor-donor pentameric ligands utilized for multimodal fluorescence microscopy of protein aggregates. ChemPhysChem **2021**, 22, 323–335.

(2) Arja, K.; Selegård, R.; Paloncýová, M.; Linares, M.; Lindgren, M.; Norman, P.; Aili, D.; Nilsson, K. P. R. Self-assembly of chiro-optical materials from nonchiral oligothiophene-porphyrin derivatives and random coil synthetic peptides. ChemPlusChem **2023**, 88, e202200262.

(3) Iakovleva, I.; Begum, A.; Pokrzywa, M.; Walfridsson, M.; Sauer-Eriksson, A. E.; Olofsson, A. The flavonoid luteolin, but not luteolin-7-O-glucoside, prevents a transthyretin mediated toxic response. PLOS ONE **2015**, 10, e0128222.

(4) Kabsch, W. XDS. Acta Crystallogr. D Biol. Crystallogr. **2010**, 66, 125–132.

(5) Winn, M. D.; Ballard, C. C.; Cowtan, K. D.; Dodson, E. J.; Emsley, P.; Evans, P. R.; Keegan, R. M.; Krissinel, E. B.; Leslie, A. G.; McCoy, A. et al. Overview of the CCP4 suite and current developments. Acta Crystallogr. D Biol. Crystallogr. **2011**, 67, 235–242.

(6) McCoy, A. J. Solving structures of protein complexes by molecular replacement with Phaser. Acta Crystallogr. D Biol. Crystallogr. **2007**, 63, 32–41.

(7) Murshudov, G. N.; Skubák, P.; Lebedev, A. A.; Pannu, N. S.; Steiner, R. A.; Nicholls, R. A.; Winn, M. D.; Long, F.; Vagin, A. A. REFMAC5 for the refinement of macromolecular crystal structures. Acta Crystallogr. D Biol. Crystallogr. **2011**, 67, 355–367.

(8) Emsley, P.; Cowtan, K. Coot: model-building tools for molecular graphics. Acta Crystallogr. D Biol. Crystallogr. **2004**, 60, 2126–2132.

(9) Frisch, M. J.; Trucks, G. W.; Schlegel, H. B.; Scuseria, G. E.; Robb, M. A.; Cheeseman, J. R.; Scalmani, G.; Barone, V.; Petersson, G. A.; Nakatsuji, H. et al. Gaussian16 Revision B.01. 2016; Gaussian Inc. Wallingford CT.

(10) Bayly, C. I.; Cieplak, P.; Cornell, W.; Kollman, P. A. A well-behaved electrostatic potential based method using charge restraints for deriving atomic charges: The RESP model. J. Phys. Chem. **1993**, 97, 10269–10280.

(11) Wang, J.; Wang, W.; Kollman, P. A.; Case, D. A. Automatic atom type and bond type perception in molecular mechanical calculations. J. Mol. Graph **2006**, 25, 247–260.

(12) Wang, J.; Wolf, R. M.; Caldwell, J. W.; Kollman, P. A.; Case, D. A. Development and testing of a general amber force field. J. Comput. Chem. **2004**, 25, 1157–1174.

(13) Todarwal, Y.; Gustafsson, C.; Thi Minh, N. N.; Ertzgaard, I.; Klingstedt, T.; Ghetti, B.; Vidal, R.; König, C.; Lindgren, M.; Nilsson, K. P. R. et al. Tau protein binding modes in Alzheimer's disease for cationic luminescent ligands. J. Phys. Chem. B . **2021**, 125, 11628–11636.

(14) Berendsen, H.; van der Spoel, D.; van Drunen, R. GROMACS: A message-passing parallel molecular dynamics implementation. Comput. Phys. Commun. **1995**, 91, 43–56.

(15) Maier, J. A.; Martinez, C.; Kasavajhala, K.; Wickstrom, L.; Hauser, K. E.; Simmerling, C. ff14SB: Improving the accuracy of protein side chain and backbone parameters from ff99SB. J. Chem. Theory Comput. **2015**, 11, 3696–3713.

(16) Eastman, P. Openmm/pdbfixer: PDBFixer fixes problems in PDB files. `https://github.com/openmm/pdbfixer`.

(17) Dennington, R.; Keith, T. A.; Millam, J. M. GaussView Version 6. 2019; Semichem Inc. Shawnee Mission KS.

(18) Jorgensen, W. L.; Chandrasekhar, J.; Madura, J. D.; Impey, R. W.; Klein, M. L. Comparison of simple potential functions for simulating liquid water. J. Chem. Phys. **1983**, 79, 926–935.

(19) Essmann, U.; Perera, L.; Berkowitz, M. L.; Darden, T.; Lee, H.; Pedersen, L. G. A smooth particle mesh Ewald method. J. Chem. Phys. **1995**, 103, 8577–8593.

(20) Hess, B.; Bekker, H.; Berendsen, H. J. C.; Fraaije, J. G. E. M. LINCS: A linear constraint solver for molecular simulations. J. Comput. Chem. **1997**, 18, 1463–1472.

(21) Torrie, G. M.; Valleau, J. P. Nonphysical sampling distributions in Monte Carlo free-energy estimation: Umbrella sampling. J. Comput. Phys. **1977**, 23, 187–199.

(22) Leach, A. Molecular Modelling: Principles and Applications, 2nd ed.; Pearson, 2001.

(23) Kästner, J. Umbrella sampling. Wiley Interdiscip. Rev. Comput. Mol. Sci. **2011**, 1, 932–942.

(24) Kumar, S.; Rosenberg, J. M.; Bouzida, D.; Swendsen, R. H.; Kollman, P. A. The weighted histogram analysis method for free-energy calculations on biomolecules. I. The method. J. Comput. Chem. **1992**, 13, 1011–1021.

(25) Hub, J. S.; de Groot, B. L.; van der Spoel, D. g_wham-a free weighted histogram analysis implementation including robust error and autocorrelation estimates. J. Chem. Theory Comput. **2010**, 6, 3713–3720.

(26) Humphrey, W.; Dalke, A.; Schulten, K. VMD: visual molecular dynamics. J. Mol. Graph. **1996**, 14, 33–38.

(27) Rurack, K.; Spieles, M. Fluorescence quantum yields of a series of red and near-infrared dyes emitting at 600- 1000 nm. Anal. Chem. **2011**, 83, 1232–1242.
